# Immediate early gene fingerprints of multi-component behaviour

**DOI:** 10.1038/s41598-019-56998-4

**Published:** 2020-01-15

**Authors:** Noemi Rook, Sara Letzner, Julian Packheiser, Onur Güntürkün, Christian Beste

**Affiliations:** 1 Department of Biopsychology, Institute of Cognitive Neuroscience, Faculty of Psychology, Ruhr University Bochum, Bochum, Germany; 2 Cognitive Neurophysiology, Department of Child and Adolescent Psychiatry, Faculty of Medicine, TU Dresden, Dresden, Germany

**Keywords:** Cognitive control, Molecular neuroscience

## Abstract

The ability to execute different responses in an expedient temporal order is central for efficient goal-directed actions and often referred to as multi-component behaviour. However, the underlying neural mechanisms on a cellular level remain unclear. Here we establish a link between neural activity at the cellular level within functional neuroanatomical structures to this form of goal-directed behaviour by analyzing immediate early gene (IEG) expression in an animal model, the pigeon (*Columba livia*). We focus on the group of zif268 IEGs and ZENK in particular. We show that when birds have to cascade separate task goals, ZENK expression is increased in the avian equivalent of the mammalian prefrontal cortex, i.e. the nidopallium caudolaterale (NCL) as well as in the homologous striatum. Moreover, in the NCL as well as in the medial striatum (MSt), the degree of ZENK expression was highly correlated with the efficiency of multi-component behaviour. The results provide the first link between cellular IEG expression and behavioural outcome in multitasking situations. Moreover, the data suggest that the function of the fronto-striatal circuitry is comparable across species indicating that there is limited flexibility in the implementation of complex cognition such as multi-component behaviour within functional neuroanatomical structures.

## Introduction

Many everyday activities involve the complex coordination of separate actions in order to generate efficient goal-directed behaviour. For example, driving a car requires us to execute different task goals such as changing gear and accelerating, while simultaneously tracking the paths of other cars in a reasonable temporal sequence. This demonstrates an example of multi-component behaviour that can be summarized as the capacity to execute, cascade and interrupt separate task goals and responses in an adequate chronological order^1–5^. From a functional neuroanatomical perspective a wide-spread prefrontal cortical^1,3–7^ and basal ganglia network^8,9^ has been implicated in this form of behaviour. While the neural correlates of multi-component behaviour are increasingly better understood in humans, their underlying mechanisms on a cellular level still remain unclear and can only be investigated using animal models and appropriate molecular neurobiological methods. Pigeons are a promising model organism, as they are capable of this form of goal-directed behaviour^10^. The question, however, remains how this behaviour can be realized on a neuronal level, as avian and mammalian brains are not one-to-one comparable^11^. If the functional neuroanatomical structures being involved in multi-component behaviour are comparable across species, pigeons would allow us to gain unique insights into the evolutionary conserved mechanisms of multi-component behaviour.

Given the involvement of the fronto-striatal network in humans^1,3,4,6–9,12,13^, it would be expected that the avian equivalents of the prefrontal cortex (PFC) and the striatum are also involved in multi-component behaviour in pigeons. An analogous structure to the mammalian PFC is the avian nidopallium caudolaterale (NCL), as they share many anatomical^14,15^, functional^16,17^, neurochemical^18,19^ and electrophysiological^16,20,21^ properties. In contrast to the mammalian PFC, the NCL is located in the caudal telencephalon^15^ and its genetic profile is different^22^, which suggests that it is unlikely that the two structures are derived from a common ancestor, but are rather the result of evolutionary convergence^23^. On the other hand, the avian basal ganglia including the striatum are considered homologues to their mammalian counterparts based on cellular, connectivity and functional data^24,25^. Based on the analogy of the NCL and the PFC and the homology of the avian and mammalian striatum, we hypothesize that both structures are involved in multi-component behaviour in pigeons. To examine this hypothesis, this study measured brain activity of the above-mentioned brain areas by quantifying immediate early gene (IEG) expression after pigeons engaged in multi-component behaviour.

IEGs are rapidly and transiently activated in response to a wide range of factors modulating cellular transcription levels^26–28^. IEGs are increasingly expressed in response to local neural activity as induced by electrical, neurobiochemical and behavioural-state changes^27,28^. Therefore, the analysis of the expression of IEGs in neural tissue can be used as a molecular neurobiological ‘imaging method’ to link neural activity within functional neuroanatomical structures to behaviour. There are several groups of IEGs^28^, such as c-fos, c-jun and zif268. Regarding the latter, zif268, homologs have been cloned from human (NGFI-A), rodents (egr-1, krox-24) and birds (ZENK)^27^. In the current study we examine ZENK expression in pigeons (*Columba livia*) after performing a STOP-CHANGE paradigm.

**Figure 1:**
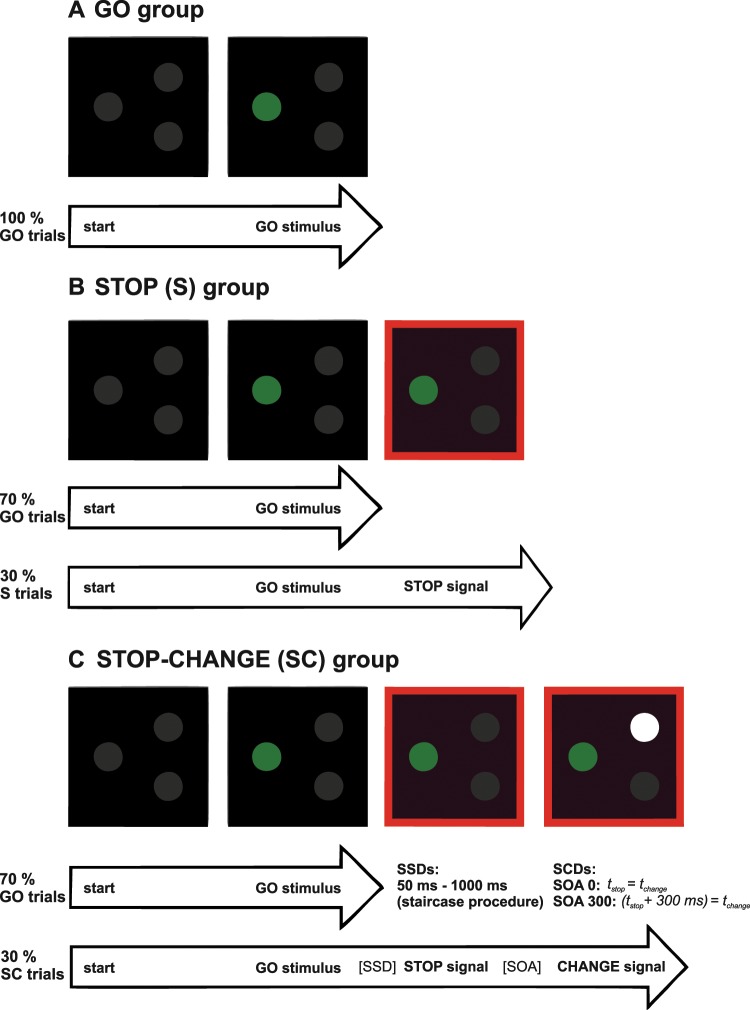
Schematic illustration of the three paradigms used for the GO, STOP and STOP-CHANGE group. (**A**) Schematic illustration of the GO paradigm. Pigeons received 100% GO trials and pecking to the GO stimulus (green circle) terminated the trial. (**B**) Schematic illustration of the STOP paradigm. In that paradigm, pigeons received 70% GO trials, where pecking the GO stimulus (green circle) terminated the trial and 30% STOP trials, where a red-light signaled that pecking had to be inhibited for 5 s to terminate the trial. (**C**) Schematic illustration of the STOP-CHANGE paradigm. This group received 70% GO trials where responding to the GO stimulus (left green circle) terminated the trial and 30% STOP-CHANGE (SC) trials where responding to the CHANGE signal (right white circle) terminated the trial. The STOP-SIGNAL delay (SSD) was adjusted by means of a staircase procedure. The STOP-CHANGE delay (SCD) between the onset of the STOP and CHANGE stimuli was fixed and set to 0 ms in half of the SC trials and to 300 ms in the other half. The CHANGE stimulus could appear at two locations (top right and bottom right circle).

A STOP-CHANGE paradigm is a way to investigate multi-component behaviour in humans as well as in pigeons^4,10^. Subjects performing this paradigm are mostly confronted with simple GO trials. However, in some trials (STOP-CHANGE trials) the GO stimulus is followed by a STOP and a CHANGE signal. These signals indicate that the GO response needs to be inhibited and a CHANGE response needs to be executed instead. This paradigm consists of two conditions that vary in the onsets of the STOP and CHANGE stimuli (STOP CHANGE delay; SCD). In the SCD 0 condition, both signals are presented at the same time, whereas in the SCD 300 condition the CHANGE stimulus is presented 300 ms after the STOP signal (Fig. 1C, see methods section for more detail). The complexity of this paradigm can vary considerably depending on whether the to-be-cascaded actions of stopping and changing are signaled at the same time, or are signaled in close succession. When trying to process two different actions at once, restricted response selection capacities^29,30^ become overstrained and multi-component behaviour unfolds inefficiently as compared to a situation where it is not necessary to process two different actions at once^4,31^. The efficacy of multi-component behaviour can be quantified by means of a single parameter (see methods section for details). We hypothesize that this efficacy parameter of multi-component is directly correlated with the brain activity in the above-mentioned structures.

## Results

### Comparison of ZENK expression between the GO, STOP and STOP-CHANGE groups

In a first step we wanted to determine which brain areas were involved in multi-component behaviour in pigeons. Therefore, pigeons were divided in three groups. The GO group only performed GO trials and served as a baseline condition controlling for movement and reward related neural activity during the task (Fig. 1A). The STOP group performed GO and STOP trials and was important to dissociate simple STOP processes from STOP-CHANGE processes (Fig. 1B). The STOP-CHANGE group performed the whole STOP-CHANGE paradigm (see methods for more details) and was the experimental group of this study in which multi-component behaviour was tested (Fig. 1C).

In the final test session, all groups received 400 trials in their particular paradigm and pigeons were perfused 60 minutes after the first trial had been started. With stainings against the immediate early gene ZENK we could quantify the contribution of the NCL, the striatum, the arcopallium and the dorsal portion of the dorsomedial hippocampus (DMd) to the above-mentioned processes. Group differences in the number of IEG-expressing neurons in all areas of interest were analyzed with a repeated measures ANOVA using the within-subject factor “area” and the between subject factor “group”.

**Figure 2:**
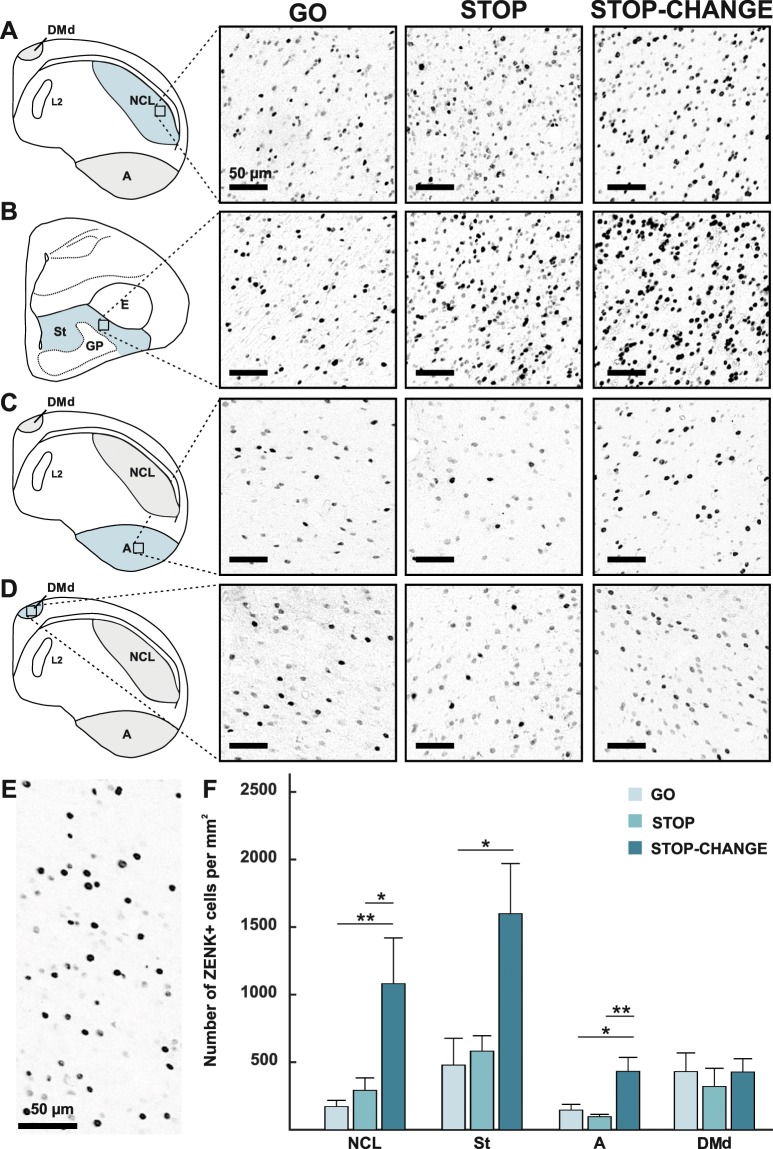
ZENK expression of all areas in the GO, STOP and STOP-CHANGE group. Schematic drawings of the (**A**) NCL, (**B**) striatum, (**C**) arcopallium and (**D**) dorsal portion of the dorsomedial hippocampus (DMd). The area of interest is highlighted in blue. The photographic images depict ZENK expression in the GO, STOP and STOP-CHANGE group in the outlined area. (**E**) Image of ZENK positive neurons under higher magnification, only the nuclei of the neurons are stained indicating antibody specificity. (**F**) Quantitative analysis of ZENK expression in NCL, striatum, arcopallium and DMd across all three groups (GO group: light blue, STOP group: darker blue, STOP-CHANGE group: dark blue). ZENK expression was significantly increased in NCL, arcopallium and striatum in the STOP-CHANGE group compared to the GO group. Furthermore, in the NCL and arcopallium ZENK expression was significantly increased in the STOP-CHANGE group compared to the STOP group. In the control area DMd no differences were found between the conditions. The error bars represent the standard error of the mean (SEM). Abbreviations: ***A***: *arcopallium*; ***DMd***: *the dorsal part of the dorsomedial hippocampus*, ***E*:** *entopallium*; ***GP****: globus pallidus*; ***NCL***: *nidopallium caudolaterale*; **St***: striatum* *p < 0.05, **p < 0.01. All scale bars represent 50 µm.

We found a main effect of area (F_(3,45)_ = 17.98, p < 0.001, η_p_^2^ = 0.545) and a main effect of group (F_(2,15)_ = 6.33, p = 0.010, η_p_^2^ = 0.458). Importantly, there was also an interaction between the factor “group” and “area” (F_(6,45)_ = 3.19, p = 0.011, η_p_^2^ = 0.298). Bonferroni corrected pairwise comparisons revealed that the NCL was significantly more active in the STOP-CHANGE group (1081 cells per mm^2^ ± 339 SEM) compared to the GO group (172 cells per mm^2^ ± 45 SEM; p = 0.009; Fig. 2A,F). Furthermore, the ZENK expression in the NCL was significantly higher in the STOP-CHANGE group as compared to the STOP group (292 cells per mm^2^ ± 92 SEM; p = 0.048; Fig. 2A,F). The ZENK expression pattern in the striatum was very similar to that of the NCL. Bonferroni corrected pairwise comparisons revealed that ZENK expression in the striatum was significantly higher in the STOP-CHANGE group (1599 cells per mm^2^ ± 370 SEM) compared to the GO group (479 cells per mm^2^ ± 198 SEM; p = 0.031; Fig. 2B,F). However, ZENK expression in the striatum in the STOP-CHANGE group was not significantly increased as compared to the STOP group (582 cells per mm^2^ ± 114 SEM; p = 0.177; Fig. 2B,F).

Another area of interest was the arcopallium which is thought to be a functional equivalent to the mammalian pre/motorcortex^32^. We analyzed this area because it receives motor input from the NCL and should be activated in tasks that require motor feedback^15^. We found a significant difference between groups within this area. The arcopallium was significantly more active in the STOP-CHANGE group (432 cells per mm^2^ ± 103 SEM) as compared to the GO group (145 cells per mm^2^ ± 42 SEM; p = 0.020; Fig. 2C,F). Furthermore, the ZENK expression in the arcopallium was significantly higher in the STOP-CHANGE group compared to the STOP group (97 cells per mm^2^ ± 17 SEM; p = 0.004; Fig. 2C,F). As the hippocampus is not expected to be involved in STOP-CHANGE processes, its subdivision DMd was analyzed as a control area to ensure that group differences were not the result of varying staining intensities. ZENK expression in DMd was similar between the STOP-CHANGE group (428 cells per mm^2^ ± 99 SEM) and the GO group (430 cells per mm^2^ ± 138 SEM; p = 1.000; Fig. 2D,F). Additionally, the activity within DMd was similar between the STOP-CHANGE group and the STOP group (320 cells per mm^2^ ± 134 SEM; p = 0.787; Fig. 2D,F). In all tested areas, the GO and the STOP group displayed similar patterns of activation (for all comparisons p = 1.000; Fig. 2F).

As already outlined above, the ANOVA also revealed a main effect of area (F_(3,45)_ = 17.980, p < 0.001) indicating that the brain areas significantly differed in their activity. However, this could easily reflect simple differences in neuron densities. To distinguish the relative importance of the NCL and the striatum that can be traced back to STOP-CHANGE processes, the relative increase in ZENK positive cells between all conditions must be considered. Interestingly, the striatum was significantly more active than the NCL in the GO group (NCL: 172 cells per mm^2^ ± 45 SEM; striatum: 479 cells per mm^2^ ± 198 SEM; p = 0.047; Fig. 2F) and STOP group (NCL: 292 cells per mm^2^ ± 92 SEM; striatum: 582 cells per mm^2^ ± 114 SEM; p = 0.049; Fig. 2F). This was however not the case in the STOP-CHANGE group (NCL: 1081 cells per mm^2^ ± 339 SEM; striatum: 1599 cells per mm^2^ ± 370 SEM; p = 0.943; Fig. 2F). The relative increase between the STOP and STOP-CHANGE group in neuronal activity that reflects the CHANGE process was 1.3 times greater for the NCL than for the striatum.

### Correlation of ZENK expression in the STOP-CHANGE group with the efficiency of multi-component behavior

**Figure 3:**
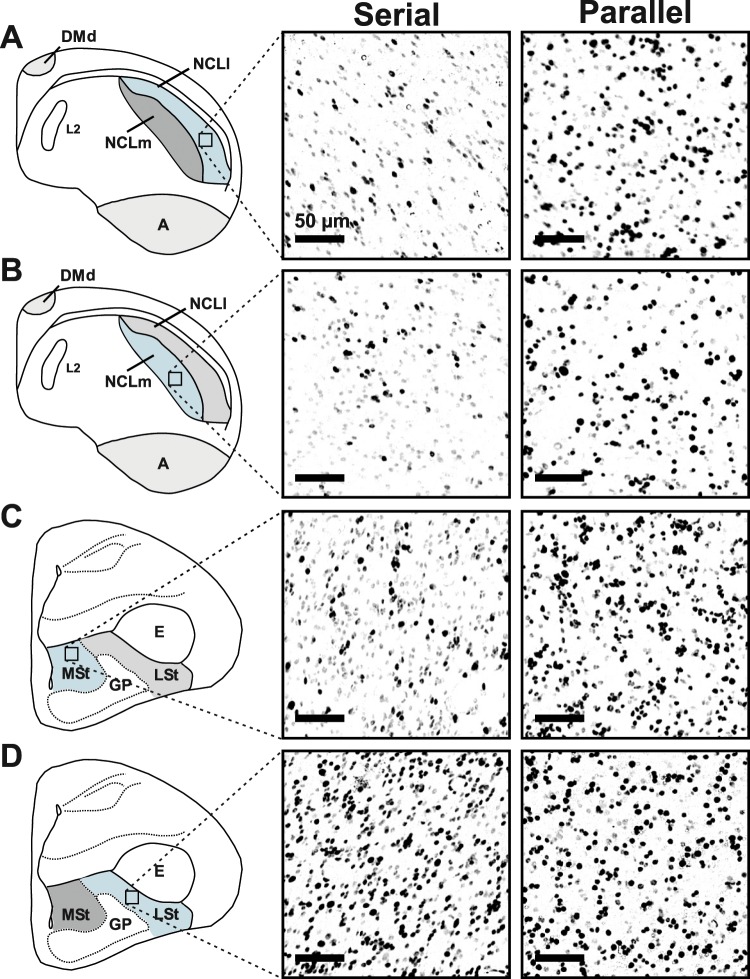
Qualitative illustration of subarea specific differences in the STOP-CHANGE group between parallel and serial processing strategies. Schematic drawings of the (**A**) NCLl, (**B**) NCLm, (**C**) MSt and (**D**) LSt. The area of interest is highlighted in blue. The photographic images depict ZENK expression in the outlined area in the STOP-CHANGE group of pigeons that used a rather serial processing strategy (left) compared to pigeons that used a rather parallel processing strategy (right). All scale bars represent 50 µm. ***A:**** arcopallium*; ***DMd:**** the dorsal part of the dorsomedial hippocampus*, ***E*:** *entopallium*; ***GP****: globus pallidus*; ***LSt:**** lateral striatum*; ***MSt:**** medial striatum*; ***NCLl:**** nidopallium caudolaterale pars lateralis*; ***NCLm****: nidopallium caudolaterale pars medialis*.

Another goal of this study was to determine whether the brain activity as measured with ZENK expression was directly correlated with the efficiency of multi-component behaviour. Therefore, we calculated an individual slope value between the CHANGE (GO2) response times (RTs) in the “SCD 0” and “SCD 300” condition for all pigeons that performed the STOP-CHANGE paradigm (for more details see method section). This slope value indicates whether the task was solved using a parallel processing strategy (slope value closer to 1, less efficient) or a serial processing strategy (slope value closer to 0, more efficient)^1,4,31^. This slope value was correlated with the number of IEG expressing neurons in all brain areas of interest. For this data analysis the NCL was subdivided into NCL pars lateralis (NCLl; Fig. 3A) and NCL pars medialis (NCLm; Fig. 3B) since both subdivisions have different neuroanatomical target regions. While the NCLm projects to the medial striatum (MSt), the NCLl projects to the arcopallium^24^. The histological data furthermore suggested to subdivide the striatum into the medial striatum (MSt, Fig. 3C) and the lateral striatum (LSt, Fig. 3D). ZENK expression in the NCLl, the NCLm and the MSt was macroscopically different between pigeons that used a rather serial processing strategy (Fig. 3A–C left) and pigeons that used a rather parallel processing strategy (Fig. 3A–C right). In contrast to this, ZENK expression could not differentiate between the processing strategies in LSt (Fig. 3D, left vs. right).

**Figure 4:**
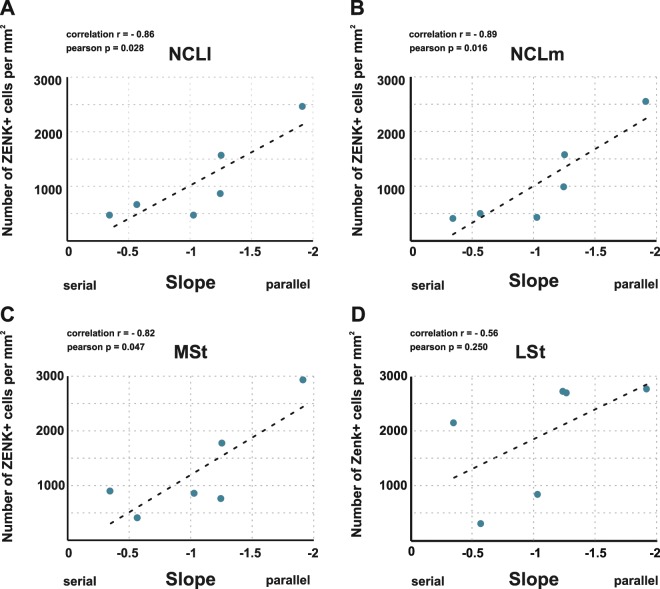
Correlation of brain activity with the efficiency of multi-component behaviour. Higher ZENK expression was associated with a steeper slope of the SCD-RT2 function (parallel processing) in (**A**) NCLl: r = −0.86, p = 0.028, (**B**) NCLm: r = −0.89, p = 0.016 and (**C**) MSt: r = − 0.82, p = 0.047. There was no significant correlation between ZENK expression and the processing strategy in (**D**) LSt: r = −0.56, p = 0.250. Abbreviations: ***LSt:**** lateral striatum*; ***MSt:**** medial striatum*; ***NCLl:**** nidopallium caudolaterale pars lateralis*; ***NCLm****: nidopallium caudolaterale pars medialis*.

We found a significant correlation between the number of ZENK-positive neurons in NCLl (r = −0.86; p = 0.028; Fig. 4A), NCLm (r = −0.89, p = 0.016, Fig. 4B) and MSt (r = −0.82, p = 0.047, Fig. 4C) with the slope values of the SCD-RT2 function. Steeper slope values, that indicated a more parallel processing strategy, were associated with a greater brain activation in all three brain areas. However, the number of ZENK-positive neurons in the LSt (r = −0.56, p = 0.250, Fig. 4D), the arcopallium (r = −0.77, p = 0.074) and DMd (r = −0.66, p = 0.150) were not significantly correlated with the slope values of the SCD-RT2 function.

## Discussion

In the current study we examined the IEG-correlates of multi-component behaviour. Thus far, previous studies in humans were able to delineate the functional neuroanatomical network and some neurobiological insights into the mechanisms of multi-component behaviour using pharmacological and genotyping approaches. However, in-depth correlates at a cellular level have remained elusive. To this end, we examined IEG expression (i.e. ZENK expression) in an animal model (i.e. pigeons), which have previously been shown to be able to display multi-component behaviour^10^. Based on the analogy of the NCL and the PFC and the homology of the avian and mammalian striatum, we hypothesized that both structures are involved in multi-component behaviour and would display increased ZENK expression compared to a simple stimulus response task as was conducted in the GO control group and compared to a pure response inhibition task as was conducted in the STOP group. Furthermore, we hypothesized that the brain activity measured in the STOP-CHANGE group within both areas would be correlated with the efficiency of multi-component behaviour. Additionally, we analysed ZENK expression in the arcopallium which is comparable to the mammalian premotor cortex^32^. Activity within all tested brain areas and groups was symmetrical between both hemispheres. While a lot of studies report behavioural and neuronal asymmetries in the avian literature^33–35^, it needs to be noted that that the basis of brain asymmetries mostly refers to timing differences between the two hemispheres^36^. However, the temporal resolution of ZENK is not fast enough to depict such timing differences. Moreover, ZENK is not sensitive enough to visualize certain aspects of lateralization as recently corroborated by an fMRI and ZENK study^37^. It is therefore still possible that aspects of multi-component behaviour are lateralized that could not be identified with ZENK. In the following, the findings will be summarized and discussed for every area separately.

Similar to several human studies providing evidence that the prefrontal cortex plays an important role^1,3–7^, we observed ZENK activity in the NCL. ZENK activity was significantly upregulated when birds performed a STOP-CHANGE paradigm, as compared to a condition with simple movement execution (GO group) and as compared to a condition with movement inhibition (STOP group). Furthermore, the ZENK expression in the NCL was significantly correlated with the efficiency of multi-component behaviour. For this correlation analysis, the NCL was subdivided into the NCL pars medialis (NCLm) and the NCL pars lateralis (NCLl). While NCLm is highly connected to MSt, resembling the fronto-striatal network in mammals^15,24^, NCLl shows strong projections to the arcopallium as a motor output structure^15,38^. Additionally, NCLl receives massive sensory input from secondary sensory areas and also projects back to these structures^15^. Both the NCLl and the NCLm revealed strong linear correlations between the number of ZENK-positive cells and the slope of the SCD-RT2 function, which provides an index of the efficacy of multi-component behaviour (see methods section for details). A steeper slope of the SCD-RT2 function has been shown to indicate less efficient multi-component behaviour (parallel processing)^2,4,31^. Thus, the data show that both subdivisions of the NCL revealed stronger activity when multi-component behaviour was less efficient. While this shows that both NCL parts are involved in multi-component behaviour, it is possible that this is due to different reasons: As the NCLm is part of a network resembling the fronto-striatal network, it is possible that this subdivision is responsible for parts of multi-component behaviour that are governed by prefrontal areas in humans^1,3,4,6–9^. Activity in the NCLl, however, could be explained by the tasks necessity to use goal-directed movements to solve this particular paradigm, as premotor units have been associated with this area^39^. Moreover, given the sensory input to NCLl, activity in this area could be a response to stimuli of different modalities involved in this task. In fact, the complexity of sensory integration processes has been shown to modulate neural processes in humans while performing an equivalent task to measure multi-component behaviour^6,7,40,41^. Furthermore, also in human studies correlations between EEG correlates and the efficiency in multi-component behaviour have been found indicating that stronger amplitudes were associated with less efficient multi-component behavior^1,2,4^.

While the results obtained from ZENK expression studies are not directly comparable to EEG amplitudes, the explanation for both findings might be similar: As outlined, the slope of the SCD-RT2 function becomes steeper whenever STOP and CHANGE stimuli are processed at the same time (i.e. in parallel). When STOP- and CHANGE-associated task goals are processed in parallel, reaction times increase because these processes must share a limited capacity. Especially the prefrontal cortex which is the mammalian equivalent to the NCL^11,23^, is subject to simultaneity constraints. The same lateral prefrontal neurons/circuits have been shown to respond to very different stimuli under different task conditions^3,42,43^. The increased activation as indicated by an increased ZENK-positive cell count might represent an attempt to process different task goals simultaneously. Overall, the ZENK expression data in the NCL indicates that this structure is important for multi-component behaviour in pigeons. As the PFC has been shown to be involved in multi-component behaviour in several human studies^1,3–7^ this finding illustrates a further functional similarity of the avian NCL to the mammalian PFC. Since the PFC and the NCL are only functionally comparable but differ in their anatomical position^15^ and genetic profile^22^, they cannot be considered homologous^23^. Thus, our data provide further evidence for the idea that the avian NCL and the mammalian PFC are a case of evolutionary convergence and that there is limited flexibility in the implementation of complex cognition such as multi-component behaviour within functional neuroanatomical structures.

Comparable to the results of the NCL, ZENK activity in the striatum was significantly upregulated when birds performed a STOP-CHANGE paradigm, as compared to a condition with simple movement execution (i.e. GO trials). However, ZENK expression in the striatum was not significantly increased as compared to a condition with motor response inhibition (STOP group). Thus, it cannot completely be ruled out that the striatal ZENK activity in the STOP-CHANGE group was the result of STOP processes rather than CHANGE processes. Nevertheless, ZENK activity in the MSt was significantly correlated with the efficiency of multi-component behaviour. This indicates that activity within this area is important for the outcome of multi-component behaviour. In contrast to this, ZENK activity in the LSt was not correlated with the efficiency of multi-component behaviour suggesting subregion-specific differences in the functionality of the avian striatum. Both, the MSt and the LSt, are thought to be homologous to the mammalian striatum (i.e. caudate and putamen) based on similar neurochemistry as well as shared hodological and developmental traits^25^. Yet, both subdivisions are not completely identical in their cellular composition and circuitry^24^. While the MSt contains cholinergic and medium-sized aspiny GABAergic interneurons that express neuropeptide Y (NPY) and somatostatin^24,44^, those cells are fewer or even absent in LSt^24,44,45^. This suggest that different neuronal computations can be performed within both striatal structures. Furthermore, while the MSt has a strong projection to the substantia nigra and projects only sparsely to the globus pallidus, the LSt shows the reversed pattern with stronger projections to the globus pallidus and only sparse projections to the substantia nigra^24,45,46^. The functional implication of this has not been investigated yet. However, it is conceivable that structures with different cell types and main targets could vary in their functionality. For example, in mammals, the substantia nigra and the globus pallidus externa belong to different subsystems. While the substantia nigra is part of the direct pathway, the globus pallidus externa is part of the indirect pathway^47^. Both pathways have been shown to have opposing functions during the movement initiation. While the direct pathway facilitates movements, the indirect pathway suppresses movements^48^. It is very likely that those two pathways could be differentially involved in multi-component behaviour. However, to make final conclusions about the relative relevance of MSt versus LSt in multi-component behaviour, future mechanistic studies are needed.

The finding that MSt was involved in the outcome of multi-component behaviour is in line with conceptual accounts suggesting that the basal ganglia medium spiny neuron system constitutes an important structure mediating response selection processes^49–52^. Furthermore, the finding is in line with several human studies showing striatal activation during multi-component behaviour^8,9,53^. Also in humans, correlations between striatal activity as measured with BOLD activation and the efficiency of multi-component behaviour have been observed, where a lower BOLD activation in the caudate nucleus was associated with inefficient (parallel) processing, and higher BOLD activation in the caudate nucleus was associated with a more efficient (serial) processing mode^9^.The authors linked this finding to the proposed role of the striatum in producing sequential representations of actions (i.e. action-chunking^54^). They argued that an increased activation of the striatum is thought to strengthen its role in action-chunking and therefore enforce a rather serial processing of cascaded actions^9^. At first glance, this correlation seems to be in the opposite direction to our findings in the MSt of pigeons, where a greater activation was associated with less efficient parallel processing. However, it needs to be noted, that results obtained from fMRI and ZENK studies are not directly comparable. BOLD reflects the overall activity within a chosen area at a specific point in time but not on a single cell level, whereas ZENK expression indicates the amount of cells that was recruited during a whole session. A possible explanation for our correlation between a high ZENK expression in MSt and a parallel/less efficient processing strategy might be that when more cells are recruited this leads to more interference and thus more inefficient/parallel processing. This result is in line with models that describe action selection as a function of multiple parallel loops running through the basal ganglia. According to those models, the most active loop dominates the selected response, whereas activity within multiple loops creates interference^51^. Taken together, the data suggests that similar to humans, striatal structures in pigeons play an important role during multi-component behaviour indicating that there are evolutionary conserved mechanisms of this behaviour.

Another finding of the study was that the ZENK expression in the arcopallium was significantly upregulated when birds performed the STOP-CHANGE paradigm, as compared to a condition with simple movement execution (GO group) and as compared to a condition with movement inhibition (STOP group). However, the activity within the arcopallium was not correlated to the efficiency of multi-component behaviour. Activity within this area might have reflected the task’s necessity to use goal-directed movements as this structure is functionally comparable to the mammalian pre/motorcortex^32^. Neurons within this area project bilaterally via the tractus occipitomesencephalicus (TOM) to brainstem nuclei to regulate body, head and beak movements^55–57^. Electrophysiological studies have found that most neurons within the arcopallium are visuomotor neurons that start firing after GO-stimulus presentation and stop firing after the animal has responded. However, those neurons are not responsive to STOP signals^36^. The finding that the STOP group displayed the lowest ZENK cell count and the STOP-CHANGE group displayed the highest ZENK cell count is well in line with this electrophysiological study. The STOP group encountered 30% STOP trials that probably did not elicit any activity in arcopallial visuomotor neurons. In contrast to this, the STOP-CHANGE group encountered 30% CHANGE trials in which two GO stimuli were presented (GO-stimulus and CHANGE-stimulus) that probably both elicited neuronal activity within different arcopallial visuomotor neurons. Thus, the activity within the arcopallium probably reflected the complexity of visuomotor behaviour that was greater in the STOP-CHANGE group as compared to the STOP and GO group. This idea is further supported by the finding that the ZENK activity within the arcopallium was not significantly correlated with the efficiency in multi-component behaviour suggesting a visuomotor rather than a cognitive mechanism.

To summarize, the current data show that comparable to human studies, the “avian PFC” as well as the MSt are involved in multi-component behaviour, and the activity in both areas is directly correlated to its efficiency indicating a similar function of the fronto-striatal circuitry between species in multi-component behaviour. With this study we furthermore provide a first step towards an appropriate animal model for future mechanistic studies in which neuronal activity can be influenced with methods such as optogenetics to investigate the direct effect of stimulation on the processing mode of multi-component behaviour.

## Materials and Methods

### Experimental subjects

For this study, N = 18 adult homing pigeons (*Columba livia*) of undetermined sex were obtained from local breeders. They were individually caged and placed on a 12-hour light-dark cycle. During the time period of training and testing, the birds were maintained at approximately 85% of their free feeding weight. All experiments were performed according to the principles regarding the care and use of animals adopted by the German Animal Welfare Law for the prevention of cruelty to animals as suggested by the European Communities Council Directive of November 24, 1986 (86/609/EEC) and were approved by the animal ethics committee of the Landesamt für Natur, Umwelt und Verbraucherschutz NRW, Germany. All efforts were made to minimize the number of animals used and to minimize their suffering.

### Skinner boxes

All experiments were conducted in conventional Skinner boxes (32 cm (w) × 34 cm (d) × 32 cm (h)). All Skinner boxes were equipped with white and red house lights and four transparent, round pecking keys (1.5 cm in diameter). Three keys were located on the front panel and one on the left side panel. A monitor was attached behind the front panel to display color stimuli behind the pecking keys. The initialization key on the side panel was illuminated by a blue LED. Below the keys on the front panel, a feeder was located, where the birds received a food reward consisting of mixed grains when responding correctly within the paradigm. A feeder light was positioned immediately above the feeder and indicated when the feeder was activated and food was available. All programs for this experiment were created using MATLAB and the Biopsychology Toolbox^58^.

### STOP-CHANGE paradigm (STOP-CHANGE group)

For this study, a STOP-CHANGE paradigm was used^10^ that reflects a direct translation of a human paradigm used in previous studies delineating the functional neuroanatomical and neurobiological correlates of multi-component behaviour in humans^1,4,6–9^. The paradigm is shown in Fig. 1C. The first phase of training consisted of an autoshaping phase, where the birds learned to associate pecking on the illuminated keys with food reward. After the pigeons had learnt to peck on all the keys, they were trained in a STOP-CHANGE paradigm with a total of 400 trials. Each trial began with the illumination of the blue initialization key on the side panel, along with the presentation of a tone, which indicated the start of a trial. After initialization, a GO stimulus (left green pecking key on the front panel) was presented after a delay of 900 ms. This delay allowed the pigeon to turn towards the front in anticipation of the GO stimulus. In 70% of trials, pecking on the GO stimulus was the correct behaviour, which was rewarded with 2 s of food reward (GO trial). In the other 30% of trials, a STOP signal was presented after the GO stimulus by turning on the red houselight. This signal indicated that the reaction to the GO stimulus had to be inhibited and a reaction to a STOP-CHANGE (SC) stimulus had to be performed. The time between the onset of the GO stimulus and onset of the STOP signal (STOP-SIGNAL delay, SSD) was initially set to 450 ms and adjusted using a staircase procedure^31^. It was modified so that the probability of successfully interrupted GO responses was 50%. If a pigeon was able to inhibit its reaction to the GO stimulus and subsequently reacted to the SC stimulus, the SSD was shortened by 50 ms for the next trial. If the animal failed to perform both actions, the SSD was prolonged by 50 ms in the next trial. The SC stimulus consisted of a white illumination of either the upper or lower pecking key to the right of the GO key. The SCD between the presentation of the STOP and the SC stimuli was 0 ms in 50% of all STOP-CHANGE trials and 300 ms in the other 50% of trials. If the pigeon correctly pecked on the SC stimulus it received a 2 s food access reward (STOP-CHANGE trial). If it incorrectly pecked on the GO stimulus at any point after the STOP signal appeared, the lights in the box turned off for 5 s. The end of each trial consisted of a 5 s inter-trial interval before the blue initialization key and a tone signaled the start of the next trial (Fig. 1C). An important aspect to consider is that the analysis of IEG in pigeons performing the paradigms reflects activities to CHANGE, STOP and usual GO processes. Therefore, it is necessary to examine pigeons in two ‘control experiments’ in which only GO or GO and STOP processes are required.

### GO group

The GO group was trained in the same Skinner boxes and performed the same autoshaping procedure as the STOP-CHANGE group (see Skinner boxes and STOP-CHANGE paradigm). This group was only trained with GO trials and served as a control group for the basic conditions of the paradigm since this condition contains all task-relevant motor executions such as pecking, eating, retrieval of the task and general movement in the Skinner box. ZENK expression patterns going beyond the ZENK expression in this control group can therefore be attributed to STOP or STOP-CHANGE related neuronal activity. The paradigm of the GO group was based on the STOP-CHANGE paradigm described above, but in this case, pigeons were only confronted with GO trials and did not have to perform STOP or STOP-CHANGE actions. Pecking on the green GO key was always rewarded with 2 s of food access (Fig. 1A).

### STOP group

The STOP group was trained in the same Skinner boxes and performed the same autoshaping procedure as the STOP-CHANGE group (see Skinner boxes and STOP-CHANGE paradigm). This group was only trained with GO and STOP trials and was important to dissociate simple STOP processes from STOP-CHANGE processes. The paradigm for the STOP group was based on the STOP-CHANGE paradigm described above. The birds in this group, however, received 70% GO and 30% STOP trials. While in GO trials pecking on the green GO stimulus was the rewarded action, in STOP trials the pigeons were rewarded for inhibiting their reaction to the GO stimulus for at least 5 seconds. In this group the STOP signal (red light) appeared always in parallel with the GO stimulus. If the birds incorrectly pecked on the GO stimulus after the STOP signal had appeared, they were punished by turning off the house lights for 5 s (Fig. 1B).

### Efficacy estimation of multi-component behaviour

Psychological models suggest that response selection is a capacity-limited process^4,5,31,59^. As outlined above, the STOP-CHANGE experiment used two different SCD intervals to present the CHANGE signal after the STOP signal. In the SCD 0 condition, STOP and CHANGE stimuli are presented at the same time, whereas in the SCD 300 condition STOP and CHANGE are presented with a 300 ms time gap. Thus, the SCD 0 condition leaves a choice how to process STOP- and CHANGE-associated processes. If the choice is to simultaneously process STOP- and CHANGE-associated task goals (i.e., in parallel), reaction times to the CHANGE stimulus (RT2) increase because these processes must share a limited capacity. However, in the SCD0 condition, it is also possible to choose a strategy in which STOP- and CHANGE-associated task goals are processed in a step-by-step (i.e., serial) manner. In this case, the STOP and CHANGE processes do not have to share a limited capacity when the STOP process is finished before the CHANGE process. This leads to shorter RT2s than the strategy in which STOP- and CHANGE-associated task goals are processed simultaneously. Critically, the SCD 300 condition always enforces a serial processing of the STOP- and CHANGE-related processes because the STOP process has finished when the CHANGE stimulus is presented 300 ms later. If such a serial processing strategy is used in the SCD 0 condition, the RT2s are comparable with those in the SCD 300 condition. The ratio of RT2 differences in the SCD 0 and SCD 300 conditions therefore gives an estimate of the strategy used during multi-component behaviour^31^.

$$slop{e}_{SCD-RT2}=\frac{{\rm{R}}{\rm{T}}2{\rm{S}}{\rm{C}}{\rm{D}}0-{\rm{R}}{\rm{T}}2{\rm{S}}{\rm{C}}{\rm{D}}300}{{\rm{S}}{\rm{C}}{\rm{D}}0-{\rm{S}}{\rm{C}}{\rm{D}}300}$$

The value becomes steeper with increasing differences between RT2SCD0 and RT2SCD300. When the STOP process has not finished by the time the CHANGE process is initiated (parallel processing strategy), the slope value becomes steeper, indicating that multi-component behaviour is less efficient. If the STOP process has finished (serial processing strategy), the slope approaches zero, which indicates that multi-component behaviour becomes more efficient^31^. Therefore, the slope of the SCD-RT2 function is flatter in the case of more efficient processing than in the case of the less efficient processing mode. The mean slope value was individually calculated for each pigeon in the final test session and correlated with the neuronal activity as measured with IEG expression.

### Activity assessment/final test session

In the final test session, all groups were trained for 400 trials in their specific paradigm (i.e. GO group, STOP group and STOP-CHANGE group). The immediate early gene ZENK has been linked to long-term memory formation and synaptic plasticity^60,61^ and was used to visualize the activity in different areas of the pigeon brain. IEGs are a useful tool to visualize brain activity since they have a low basal expression, but a fast induction and degeneration^62^. The ZENK protein for example can be detected in neurons as fast as 15 minutes after stimulation and reaches its expression peek between 1 and 2 hours after stimulation^62^. Therefore, the pigeons were sacrificed 60 minutes after the first trial of the final test session had started and when they engaged in a minimum of 80% of the total trials. Intravenous injections of equithesin (0.45 ml per 100 g body weight) were applied into the brachial vein to minimize the time variance in the uptake of the anesthetic which can occur with intramuscular injections. The perfusion started after the heart of the animal stopped beating and eyelid closure reflex was negative.

### Perfusion and tissue processing

Pigeons were perfused as previously described elsewhere^63^. The transcardial perfusion via the ventricle started with 0.9% sodium chloride (NaCl) and was followed by cold (4 °C) 4% paraformaldehyde (PFA) in 0.12 M phosphate buffer (PB; pH 7.4). After full blood exchange, the brains were removed from the skull and postfixed in 4% PFA with 30% sucrose at 4 °C for 2 hours. Hereafter, the brains were cryoprotected in 30% sucrose solution in phosphate-buffered saline (PBS; pH 7.4) for 24 hours. To simplify slicing, the brains were embedded in 15% gelatin/30% sucrose and were further fixated in 4% paraformaldehyde in PBS for 24 hours. Brains were sectioned in coronal plane in 40 µm-thick slices using a freezing microtome (Leica, Wetzlar, Germany) and stored at 4 °C in PBS with 0.1% sodium azide until further processing.

### Immunohistochemistry

For immunohistochemistry against ZENK every tenth slice of all pigeon brains were used. The staining was performed with free floating sections and the ZENK protein was visualized with a DAB (3,3 diaminobenzidinetetrahydrochloride) staining procedure. The DAB-reaction was carried out according to the experimental protocol of the used DAB-Kit (Vector Laboratories, DAB Substrate Kit SK-4100)^64^. After rinsing (3 × 10 min in PBS), the slices were incubated in 0.3% hydrogen peroxide (H_2_O_2_) in distilled water for 30 min to block endogenous peroxidases. Following further rinsing, blocking of unspecific binding sites using 10% normal horse serum (NHS; Vector Laboratories-Vectastain Elite ABC kit) in PBS with 0.3% Triton-X-100 (PBST) was performed for 30 min. In the next step, the slices were incubated with a monoclonal mouse anti-ZENK antibody (1:5000 in PBST, 7B7-A3) at 4 °C over night. The 7B7-A3 antibody was raised in mice against the ZENK peptide from the rock pigeon (*Columba livia*) and its sensitivity and selectivity for its target was verified in immunblots as well as with histological stainings^65^. The next day, the slices were rinsed in PBS (3 × 10 min) and incubated with a secondary biotinylated anti-mouse antibody (1:1000 in PBST; Vector Laboratories-Vectastain Elite ABC kit) at room temperature for 1 hour. After further rinsing (3 × 10 min in PBS), the slices were transferred into an avidin-biotin complex (Vector Laboratories-Vectastain Elite ABC kit; 1:100 in PBST). After further rinsing (3 × 10 min), slices were transferred to the DAB solution that consisted of 5 ml distilled water with 2 drops (84 µl) of buffer stock solution, 4 drops (100 µl) of DAB stock solution and 2 drops (80 µl) of nickel solution. Sections were transferred to cell wells, whereby each well contained 1 ml of the working solution. The reaction was started by adding 6 µl H_2_O_2_ solution to each well. After 2 min incubation time the slices were transferred into cell wells with PBS and rinsed (2 × 5 min in PBS). Finally, the slices were mounted on gelatin-coated slides, dehydrated in alcohol and coverslipped with depex (Fluka).

### Quantification of ZENK activity

For quantitative analysis of ZENK expression, all slices were imaged at 100× magnification using a ZEISS AXIO Imager.M1 with a camera (AxioCam MRm ZEISS 60N-C 2/3″0.63×). The whole slice was imaged bilaterally for all areas of interest. Depending on the rostro-caudal extent of the analyzed brain area, several slices could contribute to one brain region. Therefore, cells were counted in both hemispheres on four consecutive slices in NCLl, NCLm, arcopallium, MSt and LSt at different anterior (A) levels and the arithmetic mean was calculated for the further statistical analysis (planes: NCLm and NCLl: A 5.0–6.5, arcopallium: A 5.5–7.0, MSt A 9.0–10.5 and LSt A 8.0–9.5). As the hippocampus is not expected to be involved in STOP-CHANGE processes, this area was analyzed as a control area to ensure that group differences were not the result of varying staining intensities. Cells were counted in two consecutive slices in the dorsal portion of the dorsomedial hippocampus (DMd) since this subdivision displayed reliable ZENK expression in all tested animals. Cells were counted at A 5.0–6.0 and the arithmetic mean was calculated for the further statistical analysis. ZENK-positive cells were counted automatically with ImageJ (see more in the methods section ImageJ analysis) with the counter being blind to the group of the animal. The whole analysis was performed in all pigeons separately for the two hemispheres to control for hemispheric differences. A repeated measures ANOVA with the within subject factors hemisphere (left, right) and area (NCL, striatum and arcopallium) and the between subject factor group (GO, STOP, CHANGE) revealed that there was no significant difference between the hemispheres in all tested brain areas and groups (F_(__1,15)_ = 0.344, p = 0.566, η_p_^2^ = 0.022) (see also Supplementary Fig. 1). A Bayesian analysis to assess the evidence for the null hypothesis (a lack of hemispheric differences)^66^ revealed a Bayes factor of 3.45. According to Kass and Raftery^67^, this indicates substantial evidence for the null hypothesis. Therefore, the measurements from both hemispheres were pooled together for the further statistical analyses. Furthermore, for the comparison between the GO, STOP and STOP-CHANGE groups, the data from the MSt and the LSt, and the data from the NCLl and the NCLm were pooled together. The correlation analysis between ZENK activity and the processing mode in the STOP-CHANGE group was however performed for all subdivision separately.

### Image analysis

The microscopic images were processed with ImageJ and converted into an 8-bit picture. Mean staining intensities were measured within one section of the HC of all tested pigeons. A One-Way ANOVA revealed that there was no difference in mean staining intensities between all three groups (F_(2,15)_ = 0.022, p = 0.978), indicating that staining intensities were comparable between all animals. Intensely stained neurons within the striatum were taken for the upper threshold and weakly stained neurons within the HC were taken as the lower threshold. Once the threshold was determined it was kept consistent for the analysis of all images. Furthermore, the size (>20 pixel) and roundness (>0.4) of the stained particles was used as a further selection criterion and was adjusted once for all measurements. Since the whole slice had been imaged bilaterally, it was possible to delineate the whole region of interest with anatomically correct borders and count ZENK-positive cells within the whole area. All regions of interest have a different absolute size. Therefore, the size of the delineated area was always measured in ImageJ, so that the counted cells could be standardized to 1 mm^2^ in the end.

### Statistical analysis

We used the Shapiro-Wilk test to test for normal distribution of the data and the Levene’s test to test for the homogeneity of the variance. Both tests indicated that the requirements for parametric tests were violated. Therefore, we performed a logarithmic transformation of the data log_10_(x) that improved the normality as well as the equality of variances as follows:

Levene’s test before logarithmic transformation: NCL: F_(2,15)_ = 8.261, p = 0.004; striatum: F_(2,15)_ = 2.745, p = 0.096; arcopallium: F_(2,15)_ = 8.905, p = 0.003; DMd: F_(2,15)_ = 0.223, p = 0.803. Levene’s test after logarithmic transformation: NCL: F_(2,15)_ = 0.238, p = 0.791; striatum: F_(2,15)_ = 0.811, p = 0.463; arcopallium: F_(2,15)_ = 0.188, p = 0.830; DMd: F_(2,15)_ = 1.021, p = 0.384. Shapiro-Wilk test before logarithmic transformation: NCL: W_(18)_ = 0.705, p < 0.001; striatum: W_(18)_ = 0.863, p = 0.014; arcopallium: W_(18)_ = 0.775, p = 0.001; DMd: W_(18)_ = 0.835, p = 0.005. Shapiro-Wilk test after logarithmic transformation: NCL: W_(18)_ = 0.967, p = 0.731; striatum: W_(18)_ = 0.955, p = 0.507; arcopallium: W_(18)_ = 0.981, p = 0.956; DMd: W_(18)_ = 0.932, p = 0.213. Group differences in the number of IEG-expressing neurons in all areas of interest were then calculated with a repeated measures ANOVA with a within-subject factor “area” and a between-subject factor “group”. Post-hoc tests were Bonferroni corrected. Correlations between the slope of the SCD-RT2 function and the number of ZENK-positive neurons in all areas were tested with Pearson correlation test; the P-values calculated were Bonferroni corrected. Alpha was set at 0.05 for all analyses. All statistical analyses were performed with the software IBM SPSS Statistics (v. 20).

### Ethical statement

All experiments were performed according to the principles regarding the care and use of animals adopted by the German Animal Welfare Law for the prevention of cruelty to animals as suggested by the European Communities Council Directive of November 24, 1986 (86/609/EEC) and were approved by the animal ethics committee of the Landesamt für Natur, Umwelt und Verbraucherschutz NRW, Germany. All efforts were made to minimize the number of animals used and to minimize their suffering.

## Supplementary information

Supplementary Information.

## Data Availability

The data will be made available upon request.
