# Author Correction: Immediate early gene fingerprints of multi-component behaviour

**DOI:** 10.1038/s41598-020-65177-9

**Published:** 2020-07-08

**Authors:** Noemi Rook, Sara Letzner, Julian Packheiser, Onur Güntürkün, Christian Beste

**Affiliations:** 1 Department of Biopsychology, Institute of Cognitive Neuroscience, Faculty of Psychology, Ruhr University Bochum, Bochum, Germany; 2 Cognitive Neurophysiology, Department of Child and Adolescent Psychiatry, Faculty of Medicine, TU Dresden, Dresden, Germany

Correction to: *Scientific Reports* 10.1038/s41598-019-56998-4, published online 15 January 2020

The original version of this Article contained errors.

Due to an erroneous index in the MATLAB analysis script used for the behavioural analyses shown in Figure 4, the reaction times were randomly assigned to the SCD 0 and SCD 300 conditions. The authors re-did the calculations: the originally reported correlations between slope of the SCD-RT2 function and the neuronal activity are no longer statistically significant. However, the overall conclusions are qualitatively unchanged. As a result of these errors, the following has now been corrected in the Article.

In the Abstract, the following sentence has been removed:

“Moreover, in the NCL as well as in the medial striatum (MSt), the degree of ZENK expression was highly correlated with the efficiency of multi-component behaviour.”

In the Results:

“ZENK expression in the NCLl, the NCLm and the MSt was macroscopically different between pigeons that used a rather serial processing strategy (Fig. 3A–C left) and pigeons that used a rather parallel processing strategy (Fig. 3A–C right). In contrast to this, ZENK expression could not differentiate between the processing strategies in LSt (Fig. 3D, left vs. right). We found a significant correlation between the number of ZENK-positive neurons in NCLl (r =−0.86; p = 0.028; Fig. 4A), NCLm (r =−0.89, p = 0.016, Fig. 4B) and MSt (r =−0.82, p = 0.047, Fig. 4C) with the slope values of the SCD-RT2 function. Steeper slope values, that indicated a more parallel processing strategy, were associated with a greater brain activation in all three brain areas. However, the number of ZENK-positive neurons in the LSt (r =−0.56, p = 0.250, Fig. 4D), the arcopallium (r =−0.77, p = 0.074) and DMd (r =−0.66, p = 0.150) were not significantly correlated with the slope values of the SCD-RT2 function.”

now reads:

“ZENK expression in the NCLl, the NCLm and the MSt was macroscopically different between two pigeons showing extreme values on the serial-parallel processing continuum. The pigeon with the most serial processing strategy had less ZENK expression (Fig. 3A–C left) compared to the pigeon with the most parallel processing strategy (Fig. 3A–C right). In contrast to this, ZENK expression in LSt was comparable between both pigeons (Fig. 3D, left vs. right). However, we found no significant correlations between the number of ZENK-positive neurons in NCLl (ρ = 0.290, p = 0.577), NCLm (ρ = 0.257, p = 0.623), MSt (ρ = -0.257, p = 0.623), LSt (ρ = 0.429, p = 0.397), the arcopallium (ρ = 0.771, p = 0.072) and DMd (ρ = 0.486, p = 0.329) with the slope values of the SCD-RT2 function.”

“This slope value indicates whether the task was solved using a parallel processing strategy (slope value closer to 1, less efficient) or a serial processing strategy (slope value closer to 0, more efficient)^1,4,31^.”

now reads:

“This slope value indicates whether the task was solved using a parallel processing strategy (slope value closer to -1, less efficient) or a serial processing strategy (slope value closer to 0, more efficient)^1,4,31^.”

The Figure 3 legend has been corrected from:

“Qualitative illustration of subarea specific differences in the STOP-CHANGE group between parallel and serial processing strategies. Schematic drawings of the (A) NCLl, (B) NCLm, (C) MSt and (D) LSt. The area of interest is highlighted in blue. The photographic images depict ZENK expression in the outlined area in the STOP-CHANGE group of pigeons that used a rather serial processing strategy (left) compared to pigeons that used a rather parallel processing strategy (right). All scale bars represent 50 μm. A: arcopallium; DMd: the dorsal part of the dorsomedial hippocampus, E: entopallium; GP: globus pallidus; LSt: lateral striatum; MSt: medial striatum; NCLl: nidopallium caudolaterale pars lateralis; NCLm: nidopallium caudolaterale pars medialis.”

to:

“Qualitative illustration of subarea specific differences in ZENK expression in the STOP-CHANGE group between two pigeons showing extreme values on the serial-parallel processing continuum. Schematic drawings of the (A) NCLl, (B) NCLm, (C) MSt and (D) LSt. The area of interest is highlighted in blue. The photographic images depict ZENK expression in the outlined area in the STOP-CHANGE group of the pigeon using the most serial processing strategy (left column, slope: -0.04) compared to the pigeon using the most parallel processing strategy (right column, slope: -0.95). All scale bars represent 50 μm. A: arcopallium; DMd: the dorsal part of the dorsomedial hippocampus, E: entopallium; GP: globus pallidus; LSt: lateral striatum; MSt: medial striatum; NCLl: nidopallium caudolaterale pars lateralis; NCLm: nidopallium caudolaterale pars medialis.”

In addition, Figure 4 and its corresponding legend have been removed.

In the Methods:

“Correlations between the slope of the SCD-RT2 function and the number of ZENK-positive neurons in all areas were tested with Pearson correlation test;”

now reads:

“Correlations between the slope of the SCD-RT2 function and the number of ZENK-positive neurons in all areas were tested with Spearman’s correlation test;”

“If a pigeon was able to inhibit its reaction to the GO stimulus and subsequently reacted to the SC stimulus, the SSD was shortened by 50 ms for the next trial. If the animal failed to perform both actions, the SSD was prolonged by 50 ms in the next trial.”

now reads:

“If a pigeon was able to inhibit its reaction to the GO stimulus and subsequently reacted to the SC stimulus, the SSD was prolonged by 50 ms for the next trial. If the animal failed to perform both actions, the SSD was shortened by 50 ms in the next trial.”

In the Discussion:

“Furthermore, the ZENK expression in the NCL was significantly correlated with the efficiency of multi-component behaviour.”

now reads:

“Although ZENK expression was not correlated with the efficiency of multi-component behavior, ZENK expression in the NCL was macroscopically different between two pigeons showing extreme values on the serial-parallel processing continuum. The pigeon with the most serial processing strategy had less ZENK expression compared to the pigeon with the most parallel processing strategy.”

“Both the NCLl and the NCLm revealed strong linear correlations between the number of ZENK-positive cells and the slope of the SCD-RT2 function, which provides an index of the efficacy of multi-component behaviour (see methods section for details). A steeper slope of the SCD-RT2 function has been shown to indicate less efficient multi-component behaviour (parallel processing)^2,4,31^. Thus, the data show that both subdivisions of the NCL revealed stronger activity when multi-component behaviour was less efficient. While this shows that both NCL parts are involved in multi-component behaviour, it is possible that this is due to different reasons:”

now reads:

“Although both subdivisions displayed comparable numbers of ZENK positive cells, the regions might have been involved in different aspects of multi-component behaviour:”

“Furthermore, also in human studies correlations between EEG correlates and the efficiency in multi-component behaviour have been found indicating that stronger amplitudes were associated with less efficient multi-component behavior^1,2,4^.

While the results obtained from ZENK expression studies are not directly comparable to EEG amplitudes, the explanation for both findings might be similar: As outlined, the slope of the SCD-RT2 function becomes steeper whenever STOP and CHANGE stimuli are processed at the same time (i.e. in parallel). When STOP- and CHANGE-associated task goals are processed in parallel, reaction times increase because these processes must share a limited capacity. Especially the prefrontal cortex which is the mammalian equivalent to the NCL^11,23^, is subject to simultaneity constraints. The same lateral prefrontal neurons/circuits have been shown to respond to very different stimuli under different task conditions^3,42,43^. The increased activation as indicated by an increased ZENK-positive cell count might represent an attempt to process different task goals simultaneously.”

now reads:

“Additionally, human studies have found correlations between EEG correlates and the efficiency in multi-component behaviour indicating that stronger amplitudes were associated with less efficient multi-component behavior^1,2,4^. Those findings have been explained with the fact that the prefrontal cortex which is the mammalian equivalent to the NCL^11,23^ is subject to simultaneity constraints. The same lateral prefrontal neurons/circuits have been shown to respond to very different stimuli under different task conditions^3,42,43^. In our study, the absence of a correlation between the number of ZENK positive cells and the slope of the SCD-RT2 function might however have been the result of the small sample size and the poor temporal resolution of the immediate early gene ZENK. Unlike EEG, ZENK cannot depict activity differences over time within the same area but rather indicates the amount of cells that was recruited over a prolonged period of time.”

“Nevertheless, ZENK activity in the MSt was significantly correlated with the efficiency of multi-component behaviour. This indicates that activity within this area is important for the outcome of multi-component behaviour. In contrast to this, ZENK activity in the LSt was not correlated with the efficiency of multi-component behaviour suggesting subregion-specific differences in the functionality of the avian striatum.”

now reads:

“ZENK activity in both MSt and LSt was not significantly correlated with the efficiency of multi-component behavior. However, the correlation coefficients were negative for MSt and positive for LSt suggesting possible sub-region specific differences that did not reach significance due to the small sample size and the poor temporal resolution of ZENK.”

“The finding that MSt was involved in the outcome of multi-component behaviour is in line with conceptual accounts suggesting that the basal ganglia medium spiny neuron system constitutes an important structure mediating response selection processes^49,50,51,52^. Furthermore, the finding is in line with several human studies showing striatal activation during multi-component behaviour^8,9,53^. Also in humans, correlations between striatal activity as measured with BOLD activation and the efficiency of multi-component behaviour have been observed, where a lower BOLD activation in the caudate nucleus was associated with inefficient (parallel) processing, and higher BOLD activation in the caudate nucleus was associated with a more efficient (serial) processing mode^9^.”

now reads:

“In contrast to our study, human studies have observed correlations between striatal activity as measured with BOLD activation and the efficiency of multi-component behaviour, where a lower BOLD activation in the caudate nucleus was associated with inefficient (parallel) processing, and higher BOLD activation in the caudate nucleus was associated with a more efficient (serial) processing mode^9^.

“At first glance, this correlation seems to be in the opposite direction to our findings in the MSt of pigeons, where a greater activation was associated with less efficient parallel processing. However, it needs to be noted, that results obtained from fMRI and ZENK studies are not directly comparable. BOLD reflects the overall activity within a chosen area at a specific point in time but not on a single cell level, whereas ZENK expression indicates the amount of cells that was recruited during a whole session. A possible explanation for our correlation between a high ZENK expression in MSt and a parallel/less efficient processing strategy might be that when more cells are recruited this leads to more interference and thus more inefficient/parallel processing. This result is in line with models that describe action selection as a function of multiple parallel loops running through the basal ganglia. According to those models, the most active loop dominates the selected response, whereas activity within multiple loops creates interference^51^. Taken together, the data suggests that similar to humans, striatal structures in pigeons play an important role during multi-component behaviour indicating that there are evolutionary conserved mechanisms of this behaviour.”

now reads:

“Although no such correlation was found in pigeons, the differences in ZENK expression between the GO, STOP and CHANGE groups are in line with conceptual accounts suggesting that the basal ganglia medium spiny neuron system constitutes an important structure mediating response selection processes^49,50,51,52^. Furthermore, this finding indicates that similar to humans^8,9,53^, striatal structures in pigeons play an important role during multi-component behaviour suggesting that there are evolutionary conserved mechanisms of this behaviour.”

“To summarize, the current data show that comparable to human studies, the “avian PFC” as well as the MSt are involved in multi-component behaviour, and the activity in both areas is directly correlated to its efficiency indicating a similar function of the fronto-striatal circuitry between species in multi-component behaviour.”

now reads:

“To summarize, the current data show that comparable to human studies, the “avian PFC” as well as the striatum are involved in multi-component behaviour, indicating a similar functionality of the fronto-striatal circuitry across species.”

Lastly, the following sentence was removed from the Discussion:

“This idea is further supported by the finding that the ZENK activity within the arcopallium was not significantly correlated with the efficiency in multi-component behaviour suggesting a visuomotor rather than a cognitive mechanism.”

