# Supplementary material for: Immediate early gene fingerprints of multi-component behaviour
Source: Sci Rep. 2020 Jan 15;10:384. doi: 10.1038/s41598-019-56998-4 (PMC6962395; doi:10.1038/s41598-019-56998-4)
Supplement: Supplementary file 1 — Supplementary Information. [file 41598_2019_56998_MOESM1_ESM.pdf]

Immediate early gene fingerprints of multi-component behaviour

Noemi Rook[1#], Sara Letzner[1], Julian Packheiser[1], Onur Güntürkün[1], Christian Beste[2]

1 Department of Psychology, Biopsychology, Ruhr University Bochum, Germany
2 Cognitive Neurophysiology, Department of Child and Adolescent Psychiatry, Faculty of Medicine, TU Dresden, Germany

Address for correspondence
Noemi Rook
Biopsychology, Faculty of Psychology, Ruhr University Bochum, Germany
Universitätsstraße 150, 44780 Bochum, Germany
Phone: +49 234 32 21628
E-mail: noemi.rook@rub.de

Supplementary Information

The whole analysis was performed in all pigeons separately for the two hemispheres to control for hemispheric differences. A repeated measures ANOVA with the within subject factors hemisphere (left, right) and area (NCL, striatum and arcopallium) and the between subject factor group (GO, STOP, CHANGE) revealed that there was no significant difference between the hemispheres in all tested brain areas and groups $F_{(1,15)} = .344$, $p = .566$, $\eta_p^2 = .022$ (see also Supplementary Fig. 1). A Bayesian analysis to evaluate the evidence for the null hypothesis (i.e. a lack of hemispheric differences) [66] revealed a Bayes factor of 3.45. According to Kass and Raftery [67], this provides substantial evidence for the null hypothesis. Therefore, the measurements from both hemispheres were pooled together for the further statistical analyses.

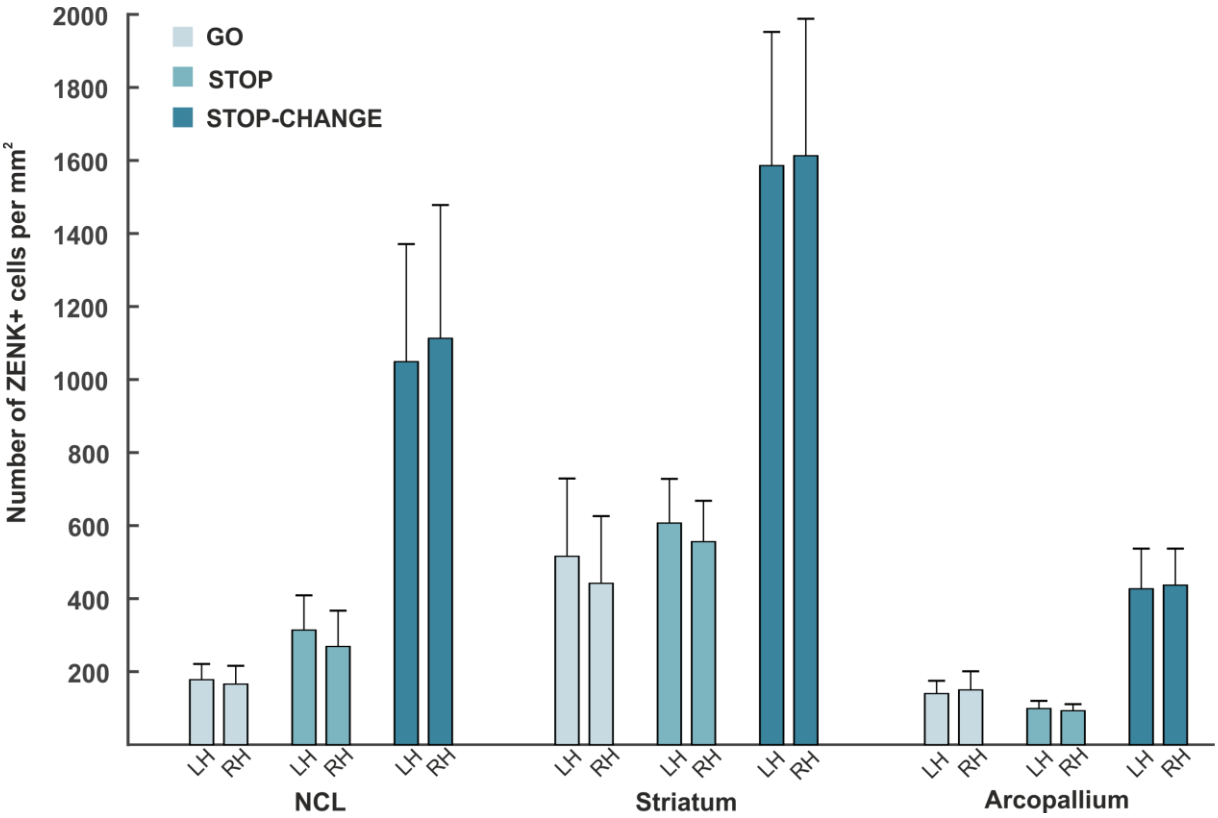

Supplementary Fig. 1 Hemisphere Comparison. Comparison of ZENK expression between the left and the right hemisphere in NCL, striatum and arcopallium in the GO (light blue), STOP (blue) and STOP-CHANGE (dark blue) group. There was no significant difference between the hemispheres in any area or group. The error bars represent the standard error of the mean (SEM).
